# Recent Advances in Renal Medullary Carcinoma

**DOI:** 10.3390/ijms23137097

**Published:** 2022-06-26

**Authors:** Yongdong Su, Andrew L. Hong

**Affiliations:** 1 Department of Pediatrics, Emory University School of Medicine, Atlanta, GA 30322, USA; ysu31@emory.edu; 2 Aflac Cancer and Blood Disorders Center, Children’s Healthcare of Atlanta, Atlanta, GA 30322, USA; 3 Winship Cancer Institute, Emory University School of Medicine, Atlanta, GA 30322, USA

**Keywords:** pediatric renal tumors, renal medullary carcinoma, SWI/SNF complex, SMARCB1, SMARCB1-deficient cancers, sickle cell trait, sickle cell disease

## Abstract

Renal medullary carcinoma (RMC) is a rare renal malignancy that has been associated with sickle hemoglobinopathies. RMC is aggressive, difficult to treat, and occurs primarily in adolescents and young adults of African ancestry. This cancer is driven by the loss of SMARCB1, a tumor suppressor seen in a number of primarily rare childhood cancers (e.g., rhabdoid tumor of the kidney and atypical teratoid rhabdoid tumor). Treatment options remain limited due in part to the limited knowledge of RMC biology. However, significant advances have been made in unraveling the biology of RMC, from genomics to therapeutic targets, over the past 5 years. In this review, we will present these advances and discuss what new questions exist in the field.

## 1. Introduction

Renal tumors in children account for 7% of all pediatric cancers [1]. In general, this group of cancers has a 5-year overall survival rate of approximately 70% [2,3]. Pediatric renal tumors include Wilms tumor (~80%) [3], renal cell carcinoma (RCC, 2–6%) [4], clear-cell sarcoma of the kidney (CCSK, 2–4%) [5], congenital mesoblastic nephroma (4%) [6], malignant rhabdoid tumor (MRT, 1.5–4%) [7], and other less common cancers such as cystic nephroma and metanephric tumors (~2%) [8]. RCC has a global incidence of about 4% [9], with a 5-year overall survival rate of approximately 78.8% [10]. Approximately 75% of RCCs are of the clear cell type (ccRCC) and about 25% of the non-clear cell carcinomas (nccRCC) [11]. nccRCC include papillary RCC (40–50% in nccRCC, 10–15 % of all RCCs), chromophobe RCC (20–30%), collecting duct carcinoma (CDC, 5–10%), renal medullary carcinoma (RMC, <5%), translocation RCC (<5%), and other (<5%) [12,13].

**Figure 1:**
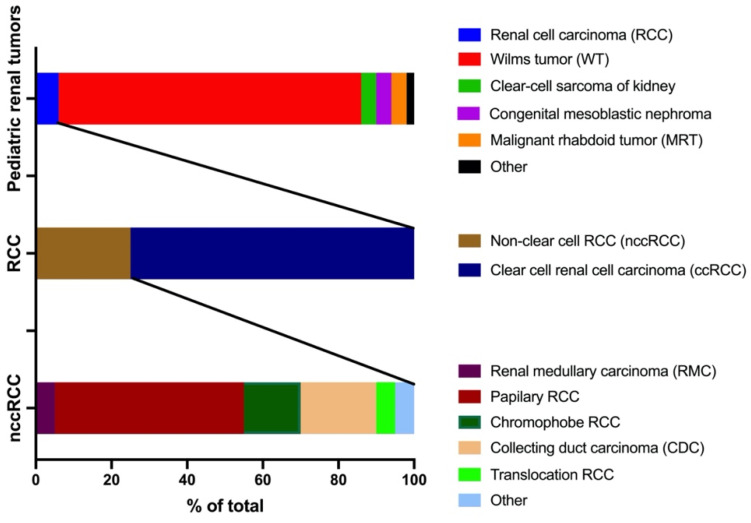
Subtypes of pediatric renal tumors and percentages.

As such, RMC accounts for 0.02–0.06% of pediatric renal tumors (Figure 1). However, there is a question of underestimation due to the age of presentation (adolescence and young adults) and capture rates within hospital systems [14]. RMC was first reported as a distinct entity by Davis et al. in 1995 [15]. This cancer mainly occurs in adolescents and young adults with sickle cell trait (SCT) or sickle cell disease (SCD) with African ancestry. The most common presenting symptoms are gross hematuria, abdominal or flank pain, decreased body mass, and abdominal masses [16]. The tumors are predominantly right-sided, ranging between 4 and 12 cm in size [17]. Most patients have metastasis at presentation occurring in regional lymph nodes, adrenal glands, lung, liver, and the peritoneum. Less commonly, metastasis has been reported on the scalp [18], brain [19], or orbit [20,21].

The prognosis of RMC is generally very poor, with a median survival time of 13 months [14], although longer survival has been occasionally reported [22,23]. One of the hallmarks of RMC is the loss of SMARCB1 (SWI/SNF-related matrix-associated actin-dependent regulator of chromatin subfamily B member 1, also known as hSNF5/INI1/BAF47) protein expression [24]. This review will focus on the recent biological advances shedding light on the mechanisms underlying RMC and the causal relationships between SCT/SCD and RMC. We will also discuss several outstanding questions in RMC, and therapeutic considerations from a biological point of view.

## 2. SMARCB1 Loss as the Primary Driver in RMC

SMARCB1 is a core subunit of the SWItch/Sucrose Non-Fermentable (SWI/SNF) complex, which is essential for a variety of cellular processes including DNA damage repair, DNA replication, proliferation, and differentiation [25]. Complete loss of SMARCB1 was initially identified in difficult-to-treat pediatric tumors such as malignant rhabdoid tumors (MRT) and atypical teratoid/rhabdoid tumors (AT/RT) [26,27]. In 1999, Stahlschmidt et al. applied FISH to a sample from an RMC patient and confirmed that RMC may be related to the translocation between chromosomes 9 and 22 [28]. In 2008, Cheng et al. compared five samples of RMC with ten samples of high-grade RCC, two urothelial carcinomas, and two pediatric renal rhabdoid tumors using immunohistochemistry (IHC). They found that all five cases of RMC showed complete IHC loss of SMARCB1, regardless of the histopathology [24].

In 2012, Calderaro et al. further studied six cases of RMC and confirmed that all six cases showed SMARCB1 loss using IHC [29]. Using high-resolution comparative genomic hybridization (CGH), they further demonstrated that RMC harbors a hemizygous deletion of *SMARCB1*: one of the *SMARCB1* alleles was completely deleted, whereas no genomic imbalance or mutation was found on the remaining allele [29]. The result was validated using multiplex ligation-dependent probe amplification (MLPA), showing hemizygous deletion of *SMARCB1* with the loss of all exons. In all RMC cases studied, they observed an upregulation of cyclin D1, indicating that the loss of SMARCB1 expression might occur during the cell cycle progression [29].

Apart from using IHC to confirm the loss of SMARCB1 expression, Liu et al. applied PCR-based microsatellite analysis in ten RMC cases and revealed a loss of heterozygosity (LOH) of *SMARCB1* [30]. Specifically, DNA of normal and tumor tissues was extracted and amplified using polymerase chain reaction (PCR). Then, a normalized allele ratio was calculated based on the following equation: LOH = (T_1_/T_2_)/(N_1_/N_2_), where T_1_, T_2_, N_1_, and N_2_ are peaks from the tumor (T) and normal (N) DNA. LOH was assumed when the ratio was less than 0.6 or more than 1.6. Nine out of ten RMC cases showed LOH of the *SMARCB1* gene; the rest showed retention of heterozygosity but the absence of SMARCB1 protein expression [30]. 

In 2016, SMARCB1 loss was again illustrated by Calderaro et al. using array comparative genomic hybridization (array CGH), whole exome sequencing (WES), and RNA sequencing (RNAseq) [31]. Five frozen samples from RMC patients were included in this research, in which four cases were associated with SCD. Array CGH suggested that the four RMC cases with SCD showed hemizygous *SMARCB1* deletion, whereas the one patient with normal hemoglobin genotype (HbAA) showed homozygous deletion. RNAseq identified *SMARCB1* fusion transcripts in all four SCD cases, which was likely a result of balanced translocation. This result was further confirmed by sequencing of genomic regions around the translocations [31].

Jia et al. expanded the study group to twenty well-characterized cases to further clarify the mechanisms of SMARCB1 loss [32]. Here, all cases studied had SCT and all tumor samples had loss of SMARCB1 based on IHC. Fluorescence in situ hybridization (FISH) analysis using three-color probes (red-orange-green) revealed that 55% of evaluated cases were concurrent hemizygous loss (absence of one set of red-orange-green signals) and translocation of *SMARCB1* (split of the remaining signals); 30% of them underwent homozygous loss of *SMARCB1* (complete loss of orange with partial loss of red and green signals), whereas the remaining 15% showed no structural or copy number alteration (diploid pattern) of *SMARCB1* [32]. A correlation between clinicopathologic and molecular features was also examined, and results indicated that homozygous deletion was favored in solid growth pattern, whereas *SMARCB1* translocation was enriched in the reticular and cribriform pattern. These results led the authors to conclude that there are different molecular mechanisms underlying the loss of SMARCB1 expression in RMC, of which biallelic inactivation is the major case [32].

The mechanism of SMARCB1 loss was further revealed and elucidated by our lab in 2019 [33]. We developed faithful RMC cell line models derived from two patients (CLF_PEDS005 and CLF_PEDS9001, respectively). Despite WES (for CLF_PEDS005) and whole genome sequencing (WGS, for CLF_PEDS9001) of primary tumor tissues showing SCT, the low purity of tumor (less than 20%) prohibited further identification of how SMARCB1 was lost due to the desmoplastic stroma. However, primary tumor cell lines (CLF_PEDS005_T1 and CLF_PEDS9001_T, respectively) and metastatic cell lines (CLF_PEDS005_T2A which grew as an adherent monolayer and CLF_PEDS005_T2B which grew in suspension) were generated. We performed FISH, WES, and WGS to compare with the results of the normal cell lines (CLF_PEDS005_N) or whole blood (CLF_PEDS9001). Dual-color break apart FISH revealed a loss of the second allele as a result of the fusion event. WGS further identified a loss-of-function intronic balanced translocation event in one of the *SMARCB1* alleles and a concurrent deletion of the other *SMARCB1* allele [33]. By using doxycycline-inducible cell lines to re-express SMARCB1, we found that re-expression of SMARCB1 in these patient-derived RMC models led to a significant decrease in cell viability, demonstrating that RMC cells depend on the loss of SMARCB1 for survival [33]. 

To further elucidate the molecular events leading to SMARCB1 loss in RMC, in 2020, Msaouel et al. performed a combination of FISH, WES and targeted sequencing, and MLPA on 38 untreated primary RMC tumor samples from a multi-institutional patient cohort [34]. Using this comprehensive genomic and transcriptomic profiling approach, they identified that 84.2% of patients with RMC harbored a genetic SMARCB1 loss. Apart from previously reported structural alterations such as recurrent loss of chromosome 22 and focal deletions of the SMARCB1 locus 22q11.23, chromosome 8q gain, where the *c-MYC* gene is located, was noted in 46.7% of RMC tumors. Accordingly, gene set enrichment analysis (GSEA) revealed that RMC tissues showed enrichment for multiple hallmark pathways associated with cell-cycle progression and DNA replication and repair, including the G2-M checkpoint, c-MYC, and E2F target genes, and TP53 and DNA repair pathways [34]. These results indicate that SMARCB1 mutations in RMC lead to high MYC expression and replicative stress [34].

## 3. Connection between RMC and Sickle Hemoglobinopathies

The most noticeable diagnostic feature of RMC is the co-occurrence of SCT, SCD, or other sickle hemoglobinopathies. Evaluation and elucidation of the correlations between SCT and RMC provide a promising pathway to bring to light the mechanism driving RMC and SMARCB1 loss. A model of SMARCB1 loss in relationship with RMC was proposed by Msaouel et al. [35]. Since the renal medulla is the most hypoxic and hypertonic environment, DNA double-strand breaks (DSB) occur frequently due to the high NaCl concentration. The regional ischemia induced by red blood cell sickling can trigger DNA repair mechanisms and induce deletions and translocations through non-homologous end joining (NHEJ). This model linked RMC and sickle hemoglobinopathies; however, further evaluation of each of the components is required to support the proposed model.

Gatalia et al. evaluated three patients with RMC (one with SCD and two without SCD) for germline and somatic mutations in genes that are commonly involved in RMC using denaturing high-performance liquid chromatography (HPLC) and direct sequencing [36]. Their results revealed that a common underlying hypoxic cellular environment is favored by RMC, as all the three cases showed increased expression of hypoxia-inducible factor (HIF). Although SCD also favors cellular hypoxia, no direct correlation between RMC and SCD could be identified [36]. 

In order to uncover the causal relationship between SCT and RMC, we applied linked-read genome sequencing to characterize the structural variants (SVs) in *SMARCB1* of RMC from 15 unrelated patients through the Children’s Oncology Group [37]. In linked-read genome sequencing, high-molecular-weight (HMW) genomic DNA is fragmented, barcoded, and amplified. A library of barcode-tagged DNA molecules then undergoes standard Illumina short-read sequencing and is computationally aligned to provide haplotype resolution [38]. This approach has advantages over short-read approaches such as WES or WGS in providing a more complete picture of the genome at haplotype resolution [39]. RNA sequencing analysis was also performed to confirm our WGS findings [37]. The haplotypes of 15 tumor samples and 12 germline samples were constructed using linked-read genome sequencing. Despite one tumor sample failing quality control in both linked-read genome sequencing and RNA-seq, biallelic somatic disruption of *SMARCB1* was detected in both parental haplotypes of all the rest tumor samples. The analysis of microhomology at DNA breaks suggested that the loss of *SMARCB1* was driven by SVs such as deletion and translocations. The presence of blunt end assembly and 1-bp microhomologies suggested that these variants disrupt *SMARCB1* function through non-homologous end joining (NHEJ) [37]. Fine-mapping of the *HBB* locus was performed to evaluate the association between RMC and SCT. Results showed that the sickle cell mutation is the strongest candidate in this region; no other allele was significantly associated with RMC [37]. This study further suggests that the primary germline mutation shared across patients with RMC remains the sickle cell trait (HbS). Furthermore, reconstruction of the haplotype in the 200 kb region surrounding *HBB* suggested that the HbS mutations were derived from three subpopulations within Africa (West, West-Central, and East Africa), indicating that there is not a founder population for the sickle cell mutation [37]. However, newer technologies such as PacBio and Oxford Nanopore long-read sequencing along with Hi-C [40], as well as recent efforts in the Telomere-to-Telomere (T2T) sequencing project and developing a repository of diverse whole genomes may provide further insights [41]. 

## 4. Therapeutic Considerations from Biology

To date, platinum-based chemotherapy is the recommended initial standard of care for patients diagnosed with RMC, given the rapid progression that can be seen in these patients [42]. This includes high-dose intensity MVAC (a combination of methotrexate, vinblastine, doxorubicin, and cisplatin) [28,43] and PCG (paclitaxel, cisplatin or carboplatin, and gemcitabine) [44,45,46], which are regimens used in urothelial carcinoma [47]. Following upfront chemotherapy, surgery is considered—particularly for those who have responded to chemotherapy. Based on small numbers, response has remained dismal, with a mortality rate of 95% [48]. One major reason for the poor outcome of chemotherapy is the poor understanding of the biology. It is urgent to develop novel treatments specific for RMC based on biological discoveries.

### 4.1. Role of Proteasome Inhibitors

We previously screened 440 compounds across a patient-derived RMC cell line and its short-term normal cell line [33]. Through genetic and pharmacologic screens in the patient-derived RMC cell lines, we identified the ubiquitin-proteasome system (UPS) as a specific vulnerability in RMC [33]. We found that upon treatment with MLN2238, we saw a G2/M arrest followed by induction of cell death pathways [49]. indicating that the UPS was a core druggable vulnerability in RMC and other SMARCB1-deficient cancers. At the same time, Carugo et al. reported that overexpression of P53 was observed in the majority of RMC cases [50]. Using embryonic mosaic mouse models of MRT, they showed that Smarcb1-deficient mouse tumors exhibit activation of the unfolded protein response (UPR) and autophagy through MYC-p19^ARF^-p53 axis [51]. Currently, the proteasome inhibitor, ixazomib, is being tested in combination with gemcitabine and doxorubicin in patients with RMC (NCT03587662) [34]. Our institution, along with others, has previously treated several patients using alternating cycles of platinum-based chemotherapy with bortezomib (a first-generation proteasome inhibitor) with a longer time to progression; however, a clinical trial is needed to validate these small numbers [52]. 

### 4.2. Other Potential Targets

Research in 2013 has demonstrated the antagonistic relationship between the SWI/SNF complex and EZH2, a catalytic subunit of the polycomb repressor complex 2 (PRC2) [53]. EZH2 was found to be upregulated in SMARCB1-deficient cancers, leading to trimethylation of lysine 27 of histone H3 (H3K27); as a result, histone and DNA were tightly bound and polycomb target genes were also broadly repressed [53]. Inhibition of EZH2 activity induced cell death in malignant rhabdoid tumor (MRT, SMARCB1-deficient cancer) cell line [54]. A phase II clinical trial is ongoing to test the efficacy of tazemetostat (EZH2 inhibitor) in adult subjects with SMARCB1-negative tumors including RMC (NCT02601950).

Beckermann et al. reported a 29-year-old African-American male RMC case with SCT who showed complete response to PD-1 inhibitor nivolumab. This case report suggested a potential anti-PD-based therapy in RMC [55]. Sodji et al. later showed that PD-L1 expression was detected in RMC patients (25% and 60% in two patients, respectively), indicating that PD-L1 is another potential target for RMC [56]. However, only a temporary response to nivolumab treatment was seen in patients with a 25% PD-L1 expression rate, with disease progression after 15 months, whereas no response was seen in the patient with higher PD-L1 expression. These results indicated that the response to nivolumab is not correlated to the level of PD-L1 expression [56]. Leruste et al. showed efficacy of anti-PD-1 monotherapy in a relevant immune-competent mouse model of ATRT, further suggesting a role of immunotherapy in SMARCB1-deficient cancers such as RMC [57]. Although promising, upfront use of immunotherapy remains experimental and should be considered only in the setting of a clinical trial.

Lipkin et al. found in one patient with RMC that there was decreased expression of ribonucleotide reductase M1 (RRM1) and phosphatase and tension homolog (PTEN) [58]. Guided by the information that PTEN deletion causes upregulation of the PI3K-AKT pathway and use of mammalian target of rapamycin (mTOR) inhibitor can block the PI3K-AKT pathway [59], the patient was treated with everolimus (mTOR inhibitor) after PCG treatment. He underwent complete remission upon everolimus treatment and remained in remission for 7 months [58]. However, the clinical trial (NCT01399918), in evaluating an mTOR inhibitor against nccRCC (two RMC patients involved), failed its primary endpoint due to both the patients with RMC showing progressive disease [60].

Msaouel et al. described how SMARCB1 loss in RMC can lead to high MYC expression that further induces high levels of DNA replication stress, and as a result, the DNA-damage repair (DDR) pathway is upregulated [34]. A DDR enzymes poly(ADP-ribose) polymerase (PARP) inhibitor can therefore be used to potentially treat RMC. Moreover, the cyclic GMP-AMP synthase interferon genes (cGAS-STING) pathway was identified as a pro-inflammatory signal of RMC [34]. Another ongoing clinical trial (NCT03274258) was activated to assess the efficacy of the cGAS-STING pathway in the immunotherapy of RMC [34,61]. 

Wiele et al. reported the use of combining the epidermal growth factor receptor (EGFR) inhibitor, erlotinib, with the vascular endothelial growth factor (VEGF) inhibitor, bevacizumab, in 10 patients with RMC based on prior studies where two patients achieved a partial response and seven patients had stable disease [34,42]. Of note, these patients were heavily pretreated and this method had been used in patients who had progressed on proteasome-inhibitor-based therapy (GDI: gemcitabine, doxorubicin, and ixazomib) [42].

## 5. Outstanding Questions in RMC Research

There are several outstanding questions. First, there were similar entities of RMC which were later found to be distinct entities of renal tumors that are not related to RMC. Secondly, there is an undefined subtype of RMC named “Unclassified renal cell carcinoma with medullary phenotype”, for patients who meet the IHC profile for RMC but do not have sickle hemoglobinopathies [62]. Its diagnosis is even harder due to the lack of sickle hemoglobinopathies. Thirdly, it has been clearly illustrated that CDC and RMC are different types of neoplasms. However, apart from similar immunobiological and histochemical properties, CDC and RMC also share typical features including SMARCB1 deficiency [63] and sickle cell hemoglobinopathies [64]. Last but not least, the generation of the PDX model and genetically engineered mouse models (GEMM) of RMC is needed to aid in biology and potential therapeutic strategies. 

### 5.1. Similar Entities of RMC (ALK Rearranged RCC)

Anaplastic lymphoma kinase (ALK) rearrangements had been found in a six-year-old male RMC patient, harboring a t(2:10)(p23;q22) translocation [65]. ALK fusion-protein analysis by mass spectrometry revealed that the N-terminal of vinculin (VCL) was fused to the C-terminal of ALK, forming a VCL-ALK fusion sequence. Sequencing of the VCL-ALK PCR product confirmed fusion of VCL exon 16 with ALK exon 20. Since ALK rearrangement was involved in various tumor types, Mariño-Enríquez et al. posited that either direct ALK inhibitors (e.g., crizotinib) or indirect ALK inhibitors (e.g., HSP90 inhibitors) could be a potential novel treatment for RMC [65]. Further research identified the ALK-rearranged RCCs (ALK-RCC) as a distinct entity that is not related to RMC [66,67]. Importantly, SMARCB1 expression was retained in ALK-RCC, which is one typical feature that is different from RMC [68].

### 5.2. Unclassified Renal Cell Carcinoma with Medullary Phenotype

“Unclassified renal cell carcinoma with medullary phenotype” was suggested by Amin et al. [62] for patients who meet the immunohistochemistry profile for RMC but do not have SCT. This entity is considerably rarer than typical RMC, occurs in older patients, and does not appear to favor the right kidney, based on the small number of cases reported so far [69]. “Unclassified RCC with medullary features” or “RCC unclassified, with medullary phenotype” (RCCU-MP) [70] mostly tends to happen in non-African ethnicities; as reported in the literature, within six Chinese patients with RMC, only one was found to have SCT [71]. It is noticeable that these Chinese patients were older than those reported African-American cases, ranging from 22 to 72 years old [71,72]. These findings are suggestive that this entity, “Unclassified renal cell carcinoma with medullary phenotype” is a variant of malignant rhabdoid tumor seen in young children. This is similar to patients with other pediatric renal tumors (e.g., Wilms tumor), presenting late in life. Future studies are needed to compare these groups.

### 5.3. Limited Mouse Models for RMC

Patient-derived xenografts (PDX) models are a useful tool for studying new anticancer agents. In 2019, Carugo et al. developed a PDX from a patient with recurrent RMC [50]. In 2020, Wei et al. developed two novel human cell line models, UOK353 and UOK360, which were derived from primary RMCs, and these had the ability to form tumors in mice [73]. These models provided a valuable tool for research and preclinical drug testing. Initial analysis of these models confirmed the potential for combination therapy of bortezomib and cisplatin in RMC and highlighted other potential therapeutic options for patients with advanced RMC [73]. This study also identified a response in these cell line models to panobinostat, an HDAC inhibitor, that could affect the gene expression dysregulation as a result of loss of SMARCB1 [73]. In 2021, Alex et al. developed the first metastatic pleural effusion (PE)-derived RMC PDX model [74]. Using this PE PDX model, sunitinib monotherapy was evaluated and demonstrated therapeutic efficacy for RMC [74]. In 2020, Msaouel et al. generated a subcutaneous RMC PDX model (RMC2X) generated from a treatment-naive sample where they identified the potential therapeutic value of targeting the PARP pathway in RMC [34]. Again, due to its rarity, no GEMM of RMC has been reported to date.

### 5.4. Distinguishing RMC from CDC

Distinguishing RMC from CDC can be challenging since RMC and CDC share overlapping morphologic and immunohistochemical features [75]. The loss of SMARCB1 was believed to be one of the typical features to distinguish RMC from CDC, yet later 15% of CDC was found in the absence of SMARCB1 expression [63]. Another typical feature for identification of RMC is the presence of SCT; however, SCT was also identified in 10% of CDC patients [64]. A recent study revealed that a notable distinction between RMC and CDC is that SMARCB1 loss in RMC activates the c-MYC pathway and subsequently induces high levels of DNA replication stress, resulting in the upregulation of DDR and cell-cycle checkpoint pathways compared with CDC [34]. Further studies are needed to better elucidate if patients with CDC with sickle cell trait are similar to patients with RMC.

### 5.5. Modifiable Risk Factors in RMC

The oncogenic driving force of RMC is believed to be *SMARCB1* loss through a primarily intronic deletion and balanced translocation. However, the identification of modifiable risk factors of RMC has rarely been reported to guide individuals with SCT to prevent the development of RMC. Shapiro et al. noticed from clinical observations that some individuals with RMC were also conducting high-intensity exercise [76]. Using a mouse model with SCT, they found that high-intensity exercise resulted in intensified renal hypoxia, especially in the right kidney [76]. Additional work in this field of study is needed.

## 6. Discussion

RMC is a rare renal tumor that affects mainly adolescents and young adults. The aggressive nature of this cancer have led clinicians to rely upon case reports and conventional chemotherapies based on therapies for RCC or TCC [77].

**Figure 2:**
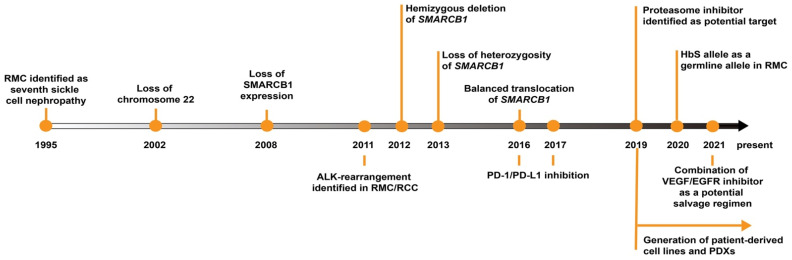
Timeline of RMC research over the past two decades.

Despite various therapies being explored and applied clinically, limited success has been seen [78]. It is, therefore, necessary to better understand the biology of RMC to help identify new therapeutic targets. More recently, a number of studies have shed light on the genomics and biology of RMC (Figure 2). The key biological and diagnostic feature of RMC is the loss of SMARCB1 protein expression. SMARCB1 is a tumor suppressor that has been characterized as absent in several pediatric tumors, such as malignant rhabdoid tumor (MRT) and atypical teratoid/rhabdoid tumor (AT/RT) [79]. The loss of SMARCB1 in MRT was referred to as “remarkably simple” since the biallelic loss of *SMARCB1* is the only mutation that caused MRT [80,81]. AT/RT, on the other hand, experienced a less than five base-pairs insertion-deletion (InDel) of *SMARCB1* [82]. Less frequently, disruption of *SMARCB1* by transposable element insertion [83] or retention of *SMARCB1* but loss of *SMARCA4* in MRTs or AT/RTs can also be detected [84]. 

The loss of SMARCB1 in RMC is comparatively more involved. It was first believed to be hemizygous deletion or LOH [29,30], but recent studies have confirmed that the majority have a deletion in one allele of SMARCB1 and a balanced translocation in the other allele [31,33,85]. However, as WGS is unable to identify the cis or trans occurrence of large stretches of variants, use of linked-read genome sequencing has enabled partial reconstruction of the genome at haplotype resolution. Data were suggestive that there was not a founder population for the sickle cell mutation and that the sickle cell trait remained an important germline risk factor [37]. 

More recent reports suggest that SMARCB1-deficient cancers are vulnerable to targeting the ubiquitin-proteasome system (UPS) [33,50]. Other therapeutic avenues reported to be used in RMC patients with varying degrees of response include platinum-based therapy [16,58], topoisomerase II inhibitors [86,87], vascular endothelial growth factor (VEGF) inhibitors including sunitinib [74,88], mTOR inhibitors such as everolimus [58,89], and immunotherapies. However, there is a clear need for development of more patient models and GEMMs to identify better therapies.

## 7. Conclusions

In summary, RMC is a rare yet lethal type of renal tumor, with loss of SMARCB1 and SCT as diagnostic features. The biological advances from next-generation sequencing have opened up our understanding of the biology of RMC. The development of several patient-derived cell lines or PDXs along with functional genomics has enabled the field to identify several potential targets for further study. These tools need to be expanded and additional funding and research are needed to identify durable cures for this rare and deadly cancer.
